# 5-Fluoro-2-methyl-3-(4-methyl­phenyl­sulfon­yl)-1-benzofuran

**DOI:** 10.1107/S1600536812001602

**Published:** 2012-01-18

**Authors:** Hong Dae Choi, Pil Ja Seo, Uk Lee

**Affiliations:** a Department of Chemistry, Dongeui University, San 24 Kaya-dong Busanjin-gu, Busan 614-714, Republic of Korea; b Department of Chemistry, Pukyong National University, 599-1 Daeyeon 3-dong, Nam-gu, Busan 608-737, Republic of Korea

## Abstract

In the title compound, C_16_H_13_FO_3_S, the 4-methyl­phenyl ring makes a dihedral angle of 76.04 (4)° with the mean plane of the benzofuran fragment. In the crystal, mol­ecules are linked by weak C—H⋯O hydrogen bonds, and by a slipped π–π inter­action between the furan and benzene rings of adjacent mol­ecules [centroid–centroid distance = 3.780 (2) Å, inter­planar distance = 3.475 (2) Å and slippage = 1.488 (2) Å].

## Related literature

For the pharmacological activity of benzofuran compounds, see: Aslam *et al.* (2009 ▶); Galal *et al.* (2009 ▶); Khan *et al.* (2005 ▶). For natural products with benzofuran rings, see: Akgul & Anil (2003 ▶); Soekamto *et al.* (2003 ▶). For the crystal structures of related compounds, see: Choi *et al.* (2010*a* ▶,*b* ▶).
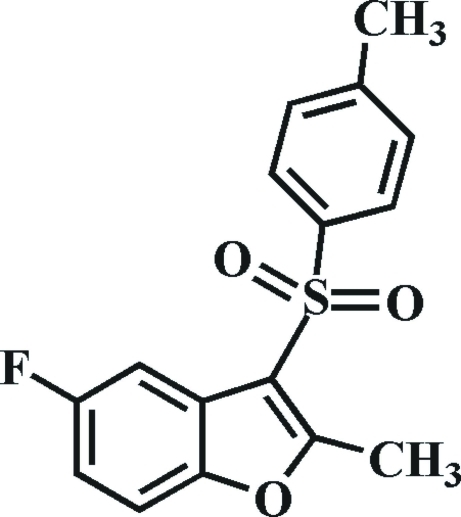


## Experimental

### 

#### Crystal data

C_16_H_13_FO_3_S
*M* *_r_* = 304.32
Monoclinic, 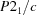

*a* = 9.9429 (6) Å
*b* = 19.7506 (11) Å
*c* = 7.3696 (4) Å
β = 104.422 (2)°
*V* = 1401.62 (14) Å^3^
*Z* = 4
Mo *K*α radiation
μ = 0.25 mm^−1^
*T* = 173 K
0.31 × 0.17 × 0.10 mm

#### Data collection

Bruker SMART APEXII CCD diffractometer
Absorption correction: multi-scan (*SADABS*; Bruker, 2009 ▶) *T*_min_ = 0.627, *T*_max_ = 0.746
12926 measured reflections
3487 independent reflections
2701 reflections with *I* > 2σ(*I*)
*R*_int_ = 0.037

#### Refinement

*R*[*F* ^2^ > 2σ(*F* ^2^)] = 0.041
*wR*(*F* ^2^) = 0.108
*S* = 1.02
3487 reflections
192 parameters
H-atom parameters constrained
Δρ_max_ = 0.41 e Å^−3^
Δρ_min_ = −0.39 e Å^−3^

### 

Data collection: *APEX2* (Bruker, 2009 ▶); cell refinement: *SAINT* (Bruker, 2009 ▶); data reduction: *SAINT* (Bruker, 2009 ▶); program(s) used to solve structure: *SHELXS97* (Sheldrick, 2008 ▶); program(s) used to refine structure: *SHELXL97* (Sheldrick, 2008 ▶); molecular graphics: *ORTEP-3* (Farrugia, 1997 ▶) and *DIAMOND* (Brandenburg, 1998 ▶); software used to prepare material for publication: *SHELXL97* (Sheldrick, 2008 ▶).

## Supplementary Material

Crystal structure: contains datablock(s) global, I. DOI: 10.1107/S1600536812001602/zj2054sup1.cif

Structure factors: contains datablock(s) I. DOI: 10.1107/S1600536812001602/zj2054Isup2.hkl

Supplementary material file. DOI: 10.1107/S1600536812001602/zj2054Isup3.cml

Additional supplementary materials:  crystallographic information; 3D view; checkCIF report

## Figures and Tables

**Table 1:** Hydrogen-bond geometry (Å, °)

*D*—H⋯*A*	*D*—H	H⋯*A*	*D*⋯*A*	*D*—H⋯*A*
C15—H15⋯O2^i^	0.95	2.58	3.246 (2)	128

Symmetry code: (i) 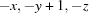.

**Fig. 1.**
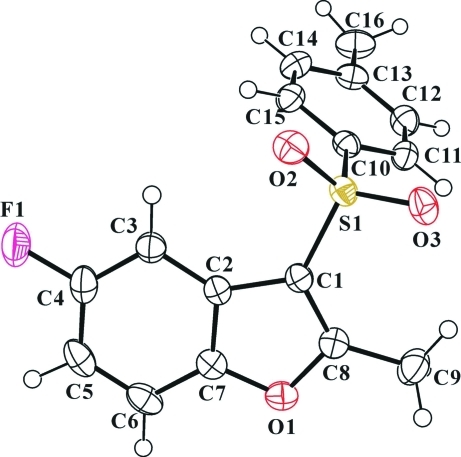
The molecular structure of the title compound with the atom numbering scheme. Displacement ellipsoids are drawn at the 50% probability level. H atoms are presented as small spheres of arbitrary radius.

**Fig. 2.**
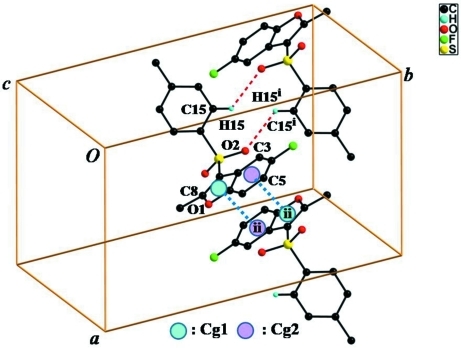
Fig. 2. A view of the C—H···O and π–π interactions (dotted lines) in the crystal structure of the title compound. H atoms non-participating in hydrogen-bonding were omitted for clarity. [Symmetry codes: (i) - x, 1- y + 1 , - z; (ii) - x + 1, - y + 1, - z.]

## supplementary crystallographic information

### Comment

Many compounds involving a benzofuran ring have drawn much attention owing to their valuable biological properties such as antibacterial and antifungal, antitumor and antiviral, and antimicrobial activities (Aslam *et al.*, 2009, Galal *et al.*, 2009, Khan *et al.*, 2005). These benzofuran derivatives occur in a wide range of natural products (Akgul & Anil, 2003; Soekamto *et al.*, 2003). As a part of our continuing study of 4-fluoro-2-methyl-1-benzofuran derivatives containing either 3-phenylsulfonyl (Choi *et al.*, 2010*a*) or 3-(4-fluorophenylsulfonyl) (Choi *et al.*, 2010*b*) substituents, we report herein the crystal structure of the title compound.

In the title molecule (Fig. 1), the benzofuran unit is essentially planar, with a mean deviation of 0.007 (1) Å from the least-squares plane defined by the nine constituent atoms. The dihedral angle between the 4-methylphenyl ring and the mean plane of the benzofurn fragment is 76.04 (4)°. The crystal packing (Fig. 2) is stabilized by weak intermolecular C—H···O hydrogen bonds between one H atom of the 4-methylphenyl ring and an oxygen of the O═S═O unit (Table 1). The crystal packing (Fig. 2) is further stabilized by a weak slipped π–π interaction between the furan and benzene rings of adjacent molecules, with a Cg1···Cg2^ii^ distance of 3.780 (2) Å and an interplanar distance of 3.475 (2) Å resulting in a slippage of 1.488 (2) Å (Cg1 and Cg2 are the centroids of the C1/C2/C7/O1/C8 furan ring and the C2–C7 benzene ring, respectively).

### Experimental

77% 3-chloroperoxybenzoic acid (560 mg, 2.5 mmol) was added in small portions to a stirred solution of 5-fluoro-2-methyl-3-(4-methylphenylsulfanyl)-1-benzofuran (326 mg, 1.2 mmol) in dichloromethane (40 mL) at 273 K. After being stirred at room temperature for 10h, the mixture was washed with saturated sodium bicarbonate solution and the organic layer was separated, dried over magnesium sulfate, filtered and concentrated at reduced pressure. The residue was purified by column chromatography (benzene) to afford the title compound as a colorless solid [yield 73%, m.p. 433–434 K; *R*_f_ = 0.48 (benzene)]. Single crystals suitable for X-ray diffraction were prepared by slow evaporation of a solution of the title compound in benzene at room temperature.

### Refinement

All H atoms were positioned geometrically and refined using a riding model, with C—H = 0.95 Å for aryl and 0.98 Å for methyl H atoms. *U*_iso_(H) =1.2*U*_eq_(C) for aryl and 1.5*U*_eq_(C)for methyl H atoms.

### Crystal data

**Table:**

C_16_H_13_FO_3_S	*F*(000) = 632
*M**_r_* = 304.32	*D*_x_ = 1.442 Mg m^−^^3^
Monoclinic, *P*2_1_/*c*	Mo *K*α radiation, λ = 0.71073 Å
Hall symbol: -P 2ybc	Cell parameters from 3987 reflections
*a* = 9.9429 (6) Å	θ = 2.4–27.6°
*b* = 19.7506 (11) Å	µ = 0.25 mm^−^^1^
*c* = 7.3696 (4) Å	*T* = 173 K
β = 104.422 (2)°	Block, colourless
*V* = 1401.62 (14) Å^3^	0.31 × 0.17 × 0.10 mm
*Z* = 4	

### Data collection

**Table:**

Bruker SMART APEXII CCD diffractometer	3487 independent reflections
Radiation source: rotating anode	2701 reflections with *I* > 2σ(*I*)
graphite multilayer	*R*_int_ = 0.037
Detector resolution: 10.0 pixels mm^-1^	θ_max_ = 28.4°, θ_min_ = 2.1°
φ and ω scans	*h* = −10→13
Absorption correction: multi-scan (*SADABS*; Bruker, 2009)	*k* = −26→25
*T*_min_ = 0.627, *T*_max_ = 0.746	*l* = −9→8
12926 measured reflections	

### Refinement

**Table:**

Refinement on *F*^2^	Primary atom site location: structure-invariant direct methods
Least-squares matrix: full	Secondary atom site location: difference Fourier map
*R*[*F*^2^ > 2σ(*F*^2^)] = 0.041	Hydrogen site location: difference Fourier map
*wR*(*F*^2^) = 0.108	H-atom parameters constrained
*S* = 1.02	*w* = 1/[σ^2^(*F*_o_^2^) + (0.0475*P*)^2^ + 0.6535*P*] where *P* = (*F*_o_^2^ + 2*F*_c_^2^)/3
3487 reflections	(Δ/σ)_max_ < 0.001
192 parameters	Δρ_max_ = 0.41 e Å^−^^3^
0 restraints	Δρ_min_ = −0.39 e Å^−^^3^

### Special details

**Table:**

Geometry. All esds (except the esd in the dihedral angle between two l.s. planes) are estimated using the full covariance matrix. The cell esds are taken into account individually in the estimation of esds in distances, angles and torsion angles; correlations between esds in cell parameters are only used when they are defined by crystal symmetry. An approximate (isotropic) treatment of cell esds is used for estimating esds involving l.s. planes.
Refinement. Refinement of F^2^ against ALL reflections. The weighted R-factor wR and goodness of fit S are based on F^2^, conventional R-factors R are based on F, with F set to zero for negative F^2^. The threshold expression of F^2^ > 2sigma(F^2^) is used only for calculating R-factors(gt) etc. and is not relevant to the choice of reflections for refinement. R-factors based on F^2^ are statistically about twice as large as those based on F, and R- factors based on ALL data will be even larger.

### Fractional atomic coordinates and isotropic or equivalent isotropic displacement parameters (Å^2^)

**Table:**

	*x*	*y*	*z*	*U*_iso_*/*U*_eq_	
S1	0.23477 (5)	0.39305 (2)	0.03088 (6)	0.02577 (13)	
F1	0.32677 (14)	0.67906 (6)	0.15349 (19)	0.0497 (3)	
O1	0.60793 (13)	0.44400 (6)	0.32052 (17)	0.0316 (3)	
O2	0.16250 (14)	0.44195 (7)	−0.10210 (17)	0.0329 (3)	
O3	0.26402 (14)	0.32735 (6)	−0.03226 (18)	0.0344 (3)	
C1	0.38972 (18)	0.43036 (9)	0.1497 (2)	0.0252 (4)	
C2	0.41125 (18)	0.50213 (9)	0.1841 (2)	0.0240 (4)	
C3	0.3330 (2)	0.56078 (9)	0.1352 (2)	0.0291 (4)	
H3	0.2397	0.5597	0.0621	0.035*	
C4	0.3993 (2)	0.62026 (9)	0.1994 (3)	0.0345 (4)	
C5	0.5348 (2)	0.62513 (10)	0.3054 (3)	0.0369 (5)	
H5	0.5739	0.6682	0.3448	0.044*	
C6	0.6127 (2)	0.56735 (10)	0.3534 (2)	0.0340 (4)	
H6	0.7062	0.5689	0.4257	0.041*	
C7	0.54778 (19)	0.50706 (9)	0.2910 (2)	0.0278 (4)	
C8	0.50969 (19)	0.39822 (9)	0.2337 (2)	0.0287 (4)	
C9	0.5545 (2)	0.32675 (10)	0.2509 (3)	0.0411 (5)	
H9A	0.5648	0.3115	0.3801	0.062*	
H9B	0.6437	0.3225	0.2179	0.062*	
H9C	0.4848	0.2988	0.1660	0.062*	
C10	0.13996 (18)	0.38170 (9)	0.2014 (2)	0.0254 (4)	
C11	0.15659 (19)	0.32275 (9)	0.3064 (2)	0.0285 (4)	
H11	0.2202	0.2889	0.2893	0.034*	
C12	0.0791 (2)	0.31405 (10)	0.4364 (3)	0.0329 (4)	
H12	0.0899	0.2736	0.5085	0.040*	
C13	−0.01424 (19)	0.36293 (10)	0.4646 (3)	0.0326 (4)	
C14	−0.0277 (2)	0.42158 (10)	0.3581 (3)	0.0344 (4)	
H14	−0.0908	0.4557	0.3755	0.041*	
C15	0.04855 (19)	0.43154 (9)	0.2272 (3)	0.0307 (4)	
H15	0.0384	0.4721	0.1558	0.037*	
C16	−0.0970 (2)	0.35188 (13)	0.6062 (3)	0.0450 (5)	
H16A	−0.0370	0.3581	0.7325	0.067*	
H16B	−0.1347	0.3058	0.5937	0.067*	
H16C	−0.1735	0.3846	0.5852	0.067*	

### Atomic displacement parameters (Å^2^)

**Table:**

	*U*^11^	*U*^22^	*U*^33^	*U*^12^	*U*^13^	*U*^23^
S1	0.0283 (2)	0.0244 (2)	0.0237 (2)	−0.00310 (18)	0.00479 (16)	−0.00129 (16)
F1	0.0602 (8)	0.0247 (6)	0.0693 (9)	0.0029 (6)	0.0258 (7)	0.0003 (6)
O1	0.0264 (7)	0.0357 (7)	0.0306 (6)	0.0000 (5)	0.0033 (5)	−0.0001 (5)
O2	0.0335 (7)	0.0364 (7)	0.0263 (6)	−0.0015 (6)	0.0026 (5)	0.0060 (5)
O3	0.0421 (8)	0.0283 (7)	0.0350 (7)	−0.0064 (6)	0.0134 (6)	−0.0088 (5)
C1	0.0263 (9)	0.0247 (9)	0.0247 (8)	−0.0013 (7)	0.0064 (7)	−0.0010 (7)
C2	0.0271 (9)	0.0236 (8)	0.0229 (8)	−0.0022 (7)	0.0090 (7)	−0.0013 (6)
C3	0.0294 (9)	0.0284 (9)	0.0316 (9)	−0.0004 (7)	0.0114 (7)	0.0001 (7)
C4	0.0460 (12)	0.0234 (9)	0.0394 (10)	0.0001 (8)	0.0206 (9)	−0.0009 (8)
C5	0.0471 (12)	0.0322 (10)	0.0360 (10)	−0.0156 (9)	0.0191 (9)	−0.0110 (8)
C6	0.0332 (10)	0.0434 (12)	0.0263 (9)	−0.0129 (9)	0.0094 (8)	−0.0087 (8)
C7	0.0296 (9)	0.0308 (10)	0.0238 (8)	−0.0024 (7)	0.0083 (7)	−0.0008 (7)
C8	0.0305 (9)	0.0289 (9)	0.0264 (8)	0.0006 (7)	0.0063 (7)	0.0000 (7)
C9	0.0422 (12)	0.0333 (11)	0.0454 (11)	0.0104 (9)	0.0062 (9)	0.0032 (9)
C10	0.0248 (9)	0.0265 (9)	0.0232 (8)	−0.0032 (7)	0.0026 (6)	−0.0004 (7)
C11	0.0312 (9)	0.0251 (9)	0.0287 (8)	0.0019 (7)	0.0063 (7)	0.0006 (7)
C12	0.0372 (10)	0.0303 (10)	0.0304 (9)	−0.0014 (8)	0.0066 (8)	0.0052 (7)
C13	0.0266 (9)	0.0426 (11)	0.0271 (9)	−0.0033 (8)	0.0041 (7)	−0.0009 (8)
C14	0.0287 (10)	0.0374 (11)	0.0373 (10)	0.0072 (8)	0.0086 (8)	0.0005 (8)
C15	0.0287 (9)	0.0286 (10)	0.0335 (9)	0.0036 (8)	0.0057 (7)	0.0047 (7)
C16	0.0340 (11)	0.0670 (16)	0.0358 (11)	−0.0017 (10)	0.0125 (9)	0.0042 (10)

### Geometric parameters (Å, °)

**Table:**

S1—O3	1.4326 (13)	C8—C9	1.476 (3)
S1—O2	1.4348 (13)	C9—H9A	0.9800
S1—C1	1.7353 (17)	C9—H9B	0.9800
S1—C10	1.7631 (18)	C9—H9C	0.9800
F1—C4	1.365 (2)	C10—C15	1.385 (3)
O1—C8	1.367 (2)	C10—C11	1.385 (2)
O1—C7	1.375 (2)	C11—C12	1.382 (3)
C1—C8	1.357 (2)	C11—H11	0.9500
C1—C2	1.447 (2)	C12—C13	1.390 (3)
C2—C3	1.392 (2)	C12—H12	0.9500
C2—C7	1.393 (2)	C13—C14	1.387 (3)
C3—C4	1.372 (3)	C13—C16	1.498 (3)
C3—H3	0.9500	C14—C15	1.381 (3)
C4—C5	1.382 (3)	C14—H14	0.9500
C5—C6	1.375 (3)	C15—H15	0.9500
C5—H5	0.9500	C16—H16A	0.9800
C6—C7	1.378 (3)	C16—H16B	0.9800
C6—H6	0.9500	C16—H16C	0.9800
			
O3—S1—O2	119.70 (8)	C8—C9—H9A	109.5
O3—S1—C1	108.78 (8)	C8—C9—H9B	109.5
O2—S1—C1	106.83 (8)	H9A—C9—H9B	109.5
O3—S1—C10	107.71 (8)	C8—C9—H9C	109.5
O2—S1—C10	107.86 (8)	H9A—C9—H9C	109.5
C1—S1—C10	105.03 (8)	H9B—C9—H9C	109.5
C8—O1—C7	107.03 (13)	C15—C10—C11	120.81 (17)
C8—C1—C2	107.68 (15)	C15—C10—S1	119.56 (13)
C8—C1—S1	126.92 (14)	C11—C10—S1	119.63 (14)
C2—C1—S1	125.35 (13)	C12—C11—C10	118.84 (17)
C3—C2—C7	119.43 (16)	C12—C11—H11	120.6
C3—C2—C1	136.18 (16)	C10—C11—H11	120.6
C7—C2—C1	104.38 (15)	C11—C12—C13	121.70 (17)
C4—C3—C2	115.71 (17)	C11—C12—H12	119.2
C4—C3—H3	122.1	C13—C12—H12	119.2
C2—C3—H3	122.1	C14—C13—C12	118.01 (17)
F1—C4—C3	117.65 (18)	C14—C13—C16	121.59 (19)
F1—C4—C5	117.49 (17)	C12—C13—C16	120.40 (18)
C3—C4—C5	124.85 (19)	C15—C14—C13	121.43 (18)
C6—C5—C4	119.67 (18)	C15—C14—H14	119.3
C6—C5—H5	120.2	C13—C14—H14	119.3
C4—C5—H5	120.2	C14—C15—C10	119.21 (17)
C5—C6—C7	116.37 (18)	C14—C15—H15	120.4
C5—C6—H6	121.8	C10—C15—H15	120.4
C7—C6—H6	121.8	C13—C16—H16A	109.5
O1—C7—C6	125.49 (17)	C13—C16—H16B	109.5
O1—C7—C2	110.52 (15)	H16A—C16—H16B	109.5
C6—C7—C2	123.97 (18)	C13—C16—H16C	109.5
C1—C8—O1	110.40 (15)	H16A—C16—H16C	109.5
C1—C8—C9	134.32 (18)	H16B—C16—H16C	109.5
O1—C8—C9	115.28 (16)		
			
O3—S1—C1—C8	−23.25 (18)	C1—C2—C7—C6	179.05 (16)
O2—S1—C1—C8	−153.76 (16)	C2—C1—C8—O1	0.03 (19)
C10—S1—C1—C8	91.85 (17)	S1—C1—C8—O1	−177.29 (12)
O3—S1—C1—C2	159.88 (14)	C2—C1—C8—C9	179.8 (2)
O2—S1—C1—C2	29.37 (17)	S1—C1—C8—C9	2.4 (3)
C10—S1—C1—C2	−85.02 (16)	C7—O1—C8—C1	0.24 (19)
C8—C1—C2—C3	178.55 (19)	C7—O1—C8—C9	−179.55 (16)
S1—C1—C2—C3	−4.1 (3)	O3—S1—C10—C15	−152.01 (14)
C8—C1—C2—C7	−0.29 (18)	O2—S1—C10—C15	−21.52 (16)
S1—C1—C2—C7	177.09 (12)	C1—S1—C10—C15	92.15 (15)
C7—C2—C3—C4	−0.3 (2)	O3—S1—C10—C11	27.24 (16)
C1—C2—C3—C4	−178.98 (19)	O2—S1—C10—C11	157.73 (14)
C2—C3—C4—F1	179.49 (15)	C1—S1—C10—C11	−88.60 (15)
C2—C3—C4—C5	0.3 (3)	C15—C10—C11—C12	0.7 (3)
F1—C4—C5—C6	−179.25 (16)	S1—C10—C11—C12	−178.54 (14)
C3—C4—C5—C6	−0.1 (3)	C10—C11—C12—C13	−0.2 (3)
C4—C5—C6—C7	−0.2 (3)	C11—C12—C13—C14	−0.2 (3)
C8—O1—C7—C6	−179.02 (17)	C11—C12—C13—C16	179.81 (18)
C8—O1—C7—C2	−0.44 (19)	C12—C13—C14—C15	0.2 (3)
C5—C6—C7—O1	178.67 (16)	C16—C13—C14—C15	−179.84 (18)
C5—C6—C7—C2	0.3 (3)	C13—C14—C15—C10	0.3 (3)
C3—C2—C7—O1	−178.64 (15)	C11—C10—C15—C14	−0.7 (3)
C1—C2—C7—O1	0.44 (18)	S1—C10—C15—C14	178.51 (14)
C3—C2—C7—C6	0.0 (3)		

### Hydrogen-bond geometry (Å, °)

**Table:**

*D*—H···*A*	*D*—H	H···*A*	*D*···*A*	*D*—H···*A*
C15—H15···O2^i^	0.95	2.58	3.246 (2)	128.

Symmetry codes: (i) −*x*, −*y*+1, −*z*.
